# Acute total aortic dissection revealed by incoercible vomiting with multiple organ failure a case report

**DOI:** 10.1016/j.amsu.2022.103518

**Published:** 2022-03-28

**Authors:** Safaa Bekkaoui, Ibtissame Ben El Mamoun, Hajar Berrichi, Noureddine Oulali

**Affiliations:** a Department of Emergency, Mohammed VI University Hospital, Oujda, Morocco; b Faculty of Medicine and Pharmacy, Mohammed I^ST^ University, Oujda, Morocco

**Keywords:** Acute aortic dissection, Multiple organ failure, Transthoracic echocardiography

## Abstract

**Introduction and importance:**

Acute aortic dissections are an uncommon entity. The different clinical manifestations especially in younger patients with no predisposing factors make it challenging to diagnose, causing delayed care and high mortality.

**Case presentation:**

In this report we describe a case of a total aortic dissection in a young man revealed by intractable vomiting with abdominal and chest pain. The dissection extended to various branches of the aorta including brachiocephalic trunk and iliac arteries, and caused multiple organ failure due to the many branches arising from the false lumen.

**Clinical disscussion:**

This case highlights the importance of considering acute aortic dissections in younger patients presenting with multi-organ failure, as well as the importance of early transthoracic echocardiography assessment in establishing the diagnosis.

**Conclusion:**

Acute aortic dissections are life threatening events, early diagnosis and management are key to prevent death especially in patients with atypical symptoms.

## Highlights

• Acute aortic dissections are uncommon among younger patients.
• Chest pain is the main symptom, but it can be revealed by other background circumstances, such as vomiting witch has not been reported earlier.
• The frozen elephant trunk repair is a hybrid approach to total aortic dissections.

## 1 Introduction

Acute aortic dissection is a dangerous condition, with an incidence estimated between 2.6 and 3.5 per 100,000 person-years [12,13].

DeBakey and the Stanford classifications are anatomical systems. The first one is based upon the origin of the tear, while the latter classifies aortic dissections on whether they involve the ascending aorta or not [14,15].

In this article, we report the case of a young patient presenting with incoercible vomiting and acute chest and abdominal pain, in whom the diagnosis of an acute total aortic dissection was established.

## 2 Case presentation

A 35 year old male presented to our emergency department with severe chest pain and abdominal pain radiating to the lower back, with intractable vomiting. The patient was symptomatic 5 h before admission.There was a history of a non documented high blood pressure and active smoking, as well as cocaine consumption, with no notable cardiovascular nor dysmorphic family history.

On admission blood pressure was 100/60 mmHg with no difference between the two arms, pulse rate was regular at 135 beats/min, oxygen saturation was 95%.

Cardiovascular assessment showed weakened heart sounds without murmurs, normal peripheral pulses, no signs of right heart failure. A 12 lead electrocardiogram showed a sinus rhythm with ST segment depression in leads DII and V4–V6 [Fig. 8].

Pulmonary assessment showed no sizzling rales.

General examination showed no clinical signs in favour of Marfan syndrome or other connective tissue disease.

**Fig. 1:**
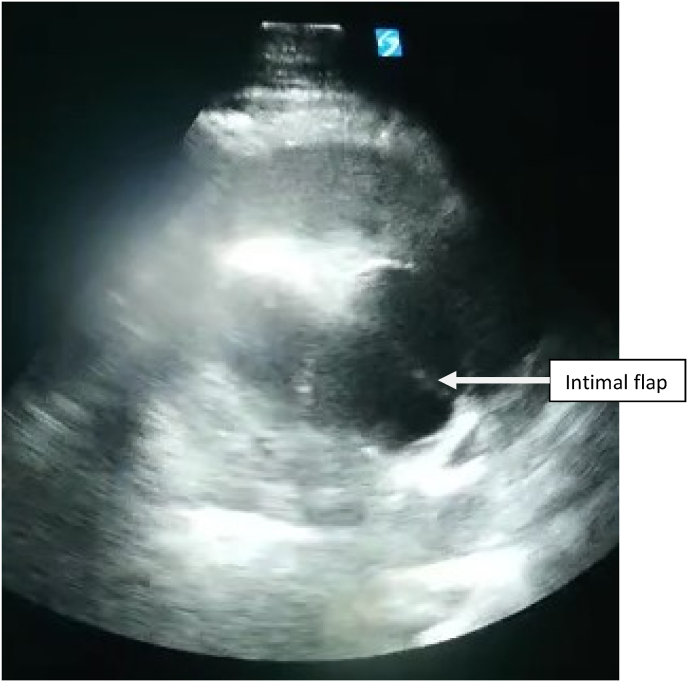
Visualisation of the intimal flap at the aortic root in TTE.

Transthoracic echocardiography was performed of technically limited quality (agitated patient). The left ventricle was hypertrophied with normal LV functions, ejection fraction was estimated at 58%. Aortic root was dilated at 45 mm, with no signs of aortic insufficiency, and an intimal flap was visualised [Fig. 1]. An average size pericardial effusion was noted without respiratory variations or right ventricular compression (20 millimiters anterior to the right ventricle, 20mm lateral to the left ventricle, 20mm facing the apex, 7mm behind the right atrium).

**Fig. 2:**
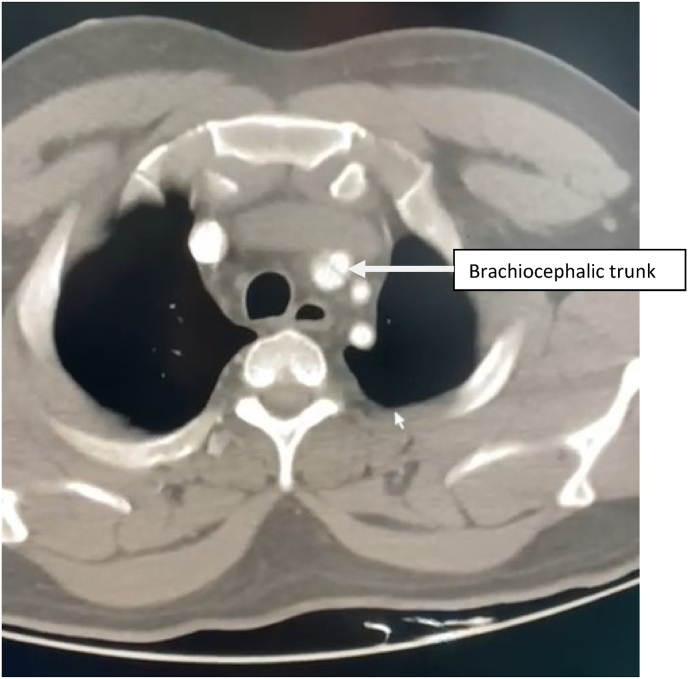
CT scan image showing the aortic dissection extending to the brachiocephalic trunk.

**Fig. 3:**
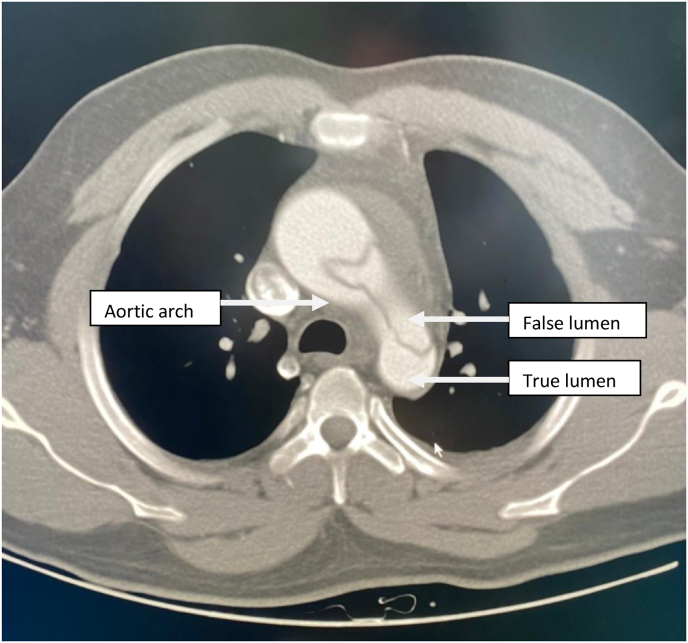
a CT scan image showing the dissection in the aortic arch (c) with the true (b) and the false lumen (a).

**Fig. 4:**
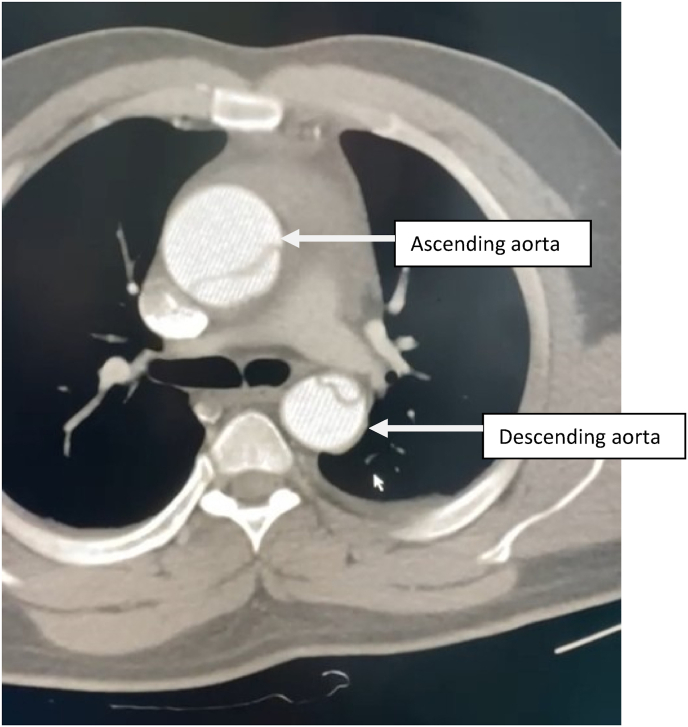
CT scan image showing the dissection in the ascending and the descending aorta.

**Fig. 5:**
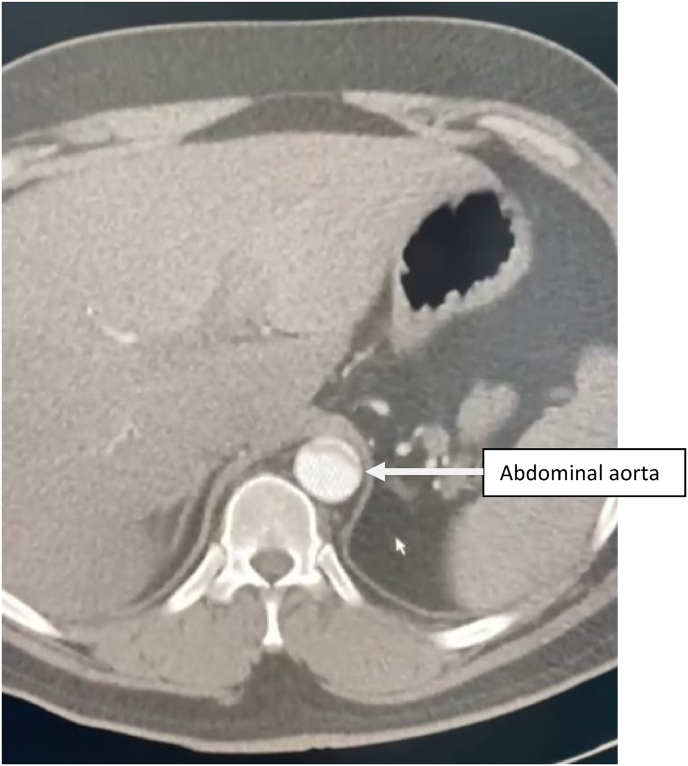
CT scan image showing the extension of the dissection to the abdominal aorta.

**Fig. 6:**
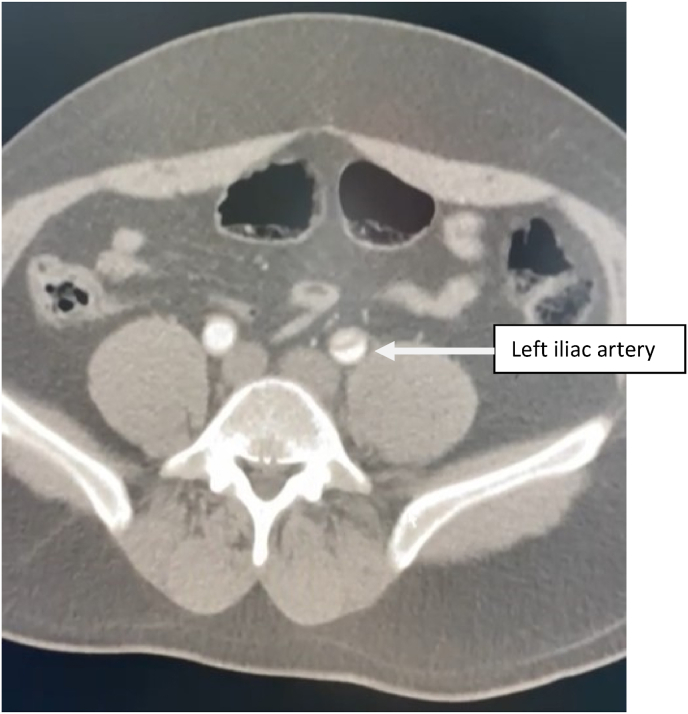
CT scan image showing the extension of the dissection to the iliac arteries.

**Fig. 7:**
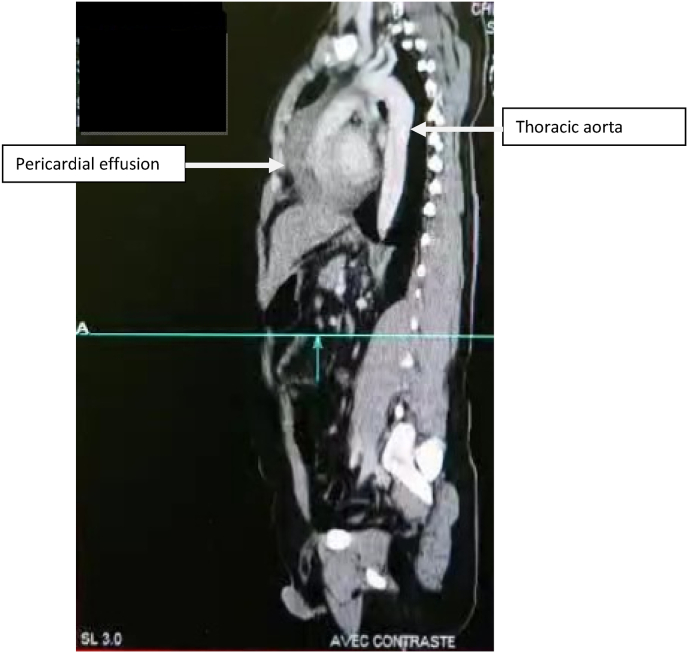
Coronal view of the dissection with pericardial effusion.

Contrast enhanced thoracic and abdominal CT performed in extreme emergency demonstrated a Stanford A and Debakey type I aortic dissection [Figs. 3 and Fig. 4, Fig. 5, Fig. 6, Fig. 7] extending to the brachiocephalic trunk [Fig. 2], right iliac common artery [Fig. 6], and right above the bifurcation of the left iliac artery.

**Fig. 8:**
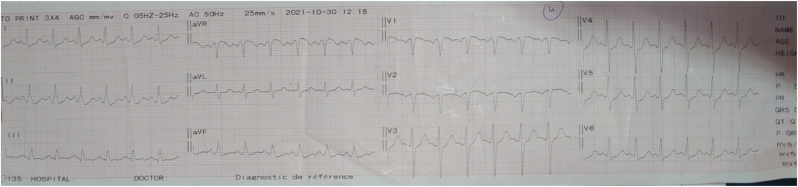
Electrocardiogram

Right coronary and carotid artery, visceral branches of the abdominal aorta, including superior mesenteric artery, celiac trunk, right renal artery, and inferior mesenteric artery all arised from the false lumen.

A parietal thrombus of the sub-renal aorta and the left iliac artery was individualized, as well as an infarct of a segment in the right liver.

Laboratory results revealed normal hemoglobin [12.8 g/dL; 13–18], leukocytosis [18.390/mL; 4.0–11.000/mL], normal platelet count [168.600/mL; 150–400.000 mL], elevated AST (aspartate transaminase) [11031 IU/L; 5–34 IU/L], normal ALT (alanine transaminase) [6 IU/L; 0–55 IU/L], normal lipase levels [116 UI/L; 4–76 UI/L], creatinine levels increased within 24 hours [from 28.09 to 53.56 mg/L; 7,2–12,5 mg/dL], BUN (blood urea nitrogen) [0.57–1.15 g/L; 0,15–0,45 g/L], elevated cardiac troponin [600 ng/L; <26 ng/L], CK [498 IU/L; 35–232 IU/L], normal activated partial thromboplastin time ratio, and a hypokalemia related to vomiting [2.9 mmol/L; 3.1–5.1].

We set up a blood pressure monitoring in both arms, as well as heart rate and pulse oxymetry monitors. The patient had risen his systolic blood pressure to 140 mmHg, so we introduced morphine, as well as intravenous nicardipine infusion starting at 5mg/hour to maintain a systolic blood pressure between 100 and 120 mmHg. We corrected his hypokalemia with intravenous potassium, and administered anti-emetic drugs.

In collaboration with the closest cardiothoracic center, we transferred the patient for emergency surgical treatment 30 hours after admission due to logistic limitations.

The patient underwent a « frozen elephant trunk repair » and died after surgery.

## 3 Discussion

Most often, aortic dissections are caused by an intimal tear and a laceration of the inner layer of the aortic media, allowing blood to enter and split the aortic media, creating a true and a false lumen [1].

The false lumen expands to balance between the wall tension and the aortic pressure, consequently causing true lumen collapse, malperfusion of aortic branches, and end organ ischemia [2].

According to the Mehta et al. study, patients with advanced age over 70 are most often patients with atherosclerosis, aortic aneurysms, intramural hematomas, and iatrogenic dissections [3], while younger patients are more likely to have a genetic component, eg Marfan syndrome which represents 8.5% of patients around 55 years with aortic dissections [3]. Moreover, substances capable of inducing hypertension may precipitate acute aortic dissections (eg cocaine) [4,5], which may have been the case of our patient.

As reported by Peter et al., 80–90% of acute aortic dissections manifest as severe acute chest or back pain [7]. The pain is most often described as sharp rather than tearing or reaping. It is important to mention that radiating pain may point to an anterograde or retrograde extension of the dissection, potentially extending to visceral branches of the abdominal aorta. Our patient presented abdominal pain and incoercible vomiting. As far as we know, vomiting has never been reported as a symptom of acute aortic dissection.

Hypotension is most often described in type A aortic dissections while hypertension is typical in type B dissections [7].

Electrocardiography could be normal in 30% of cases, or showST-T wave changes in 40% [7,8]. Our patient presented ST segment depression. For imaging modalities, TTE is considered the first-line method. CT angiography and MRI are used to determine the diagnosis, classify the location and extent of the dissection. The definitive diagnosis is established by imaging that demonstrates two distinct lumens separated by an intimal flap as was the case with our patient who showed extensive aortic dissection going all the way to its iliac branches [9].

Once the acute aortic dissection is confirmed, anti-impulse therapy using beta-blockers and pain control using morphine should be initiated [10].

Systolic blood pressure should be decreased, using esmolol, to the lowest level tolerated, generally between 100 and 120 mmHg. In our patient's case, nicardipine was used due to the lack of resources [9,10].

Type A aortic dissections are treated with early surgery, taking into account the patient's comorbidities [9,10]. Whereas type B aortic dissections are usually treated with ongoing medical treatment, with the endovascular approach indicated in patients who present a complication or a progressive dissection [10].

The “frozen elephant trunk repair” is a hybrid approach that uses open surgery to repair the ascending aorta, while using a stent-graft to replace the descending aorta [11,12].

According to Mehta RH et al. study, factors predicting bad prognosis include advanced age, acute chest pain, hemodynamic instability, tamponade, ischemic injuries, and neurological impairment [13].

This work has been reported in line with the SCARE 2020 criteria [16].

## 4 Conclusion

Medical management is initiated for all types of aortic dissection once the diagnosis has been made and can be the only established treatment in some patients. Whereas surgical intervention, with ongoing medical management, is indicated for type A acute aortic dissection as well as complicated type B acute aortic dissection.

## Ethical approval

This is a case report that doesn't need an ethical approval.

## Funding sources

None.

## Author contribution

Dr Bekkaoui Safaa: writing the paper, data collection, Dr Ben el Mamoun Ibtissame: writing the paper, data collection, Dr Berrichi Hajar: writing the paper, data collection.

## Research registration

This is a case report that doesn't need an research registration.

## Consent

Written informed consent was obtained from the patient for publication of this case report and accompanying images. A copy of the written consent is available for review by the Editor-in-Chief of this journal on request.

## Guarantor

Dr Bekkaoui Safaa.

## Provenance and peer review

Not commissioned, externally peer reviewed.

## Declaration of competing interest

None declared.
